# Radiological assessment of shoulder balance following posterior spinal fusion for thoracic adolescent idiopathic scoliosis

**DOI:** 10.1186/1748-7161-10-S2-S18

**Published:** 2015-02-11

**Authors:** Takashi Namikawa, Akira Matsumura, Minori Kato, Kazunori Hayashi, Hiroaki Nakamura

**Affiliations:** 1 Department of Orthopedic Surgery, Osaka City General Hospital, Osaka 534-0021, Japan; 2 Department of Orthopedic Surgery, Osaka City University Graduate School of Medicine, Osaka 534-0021, Japan

**Keywords:** adolescent idiopathic scoliosis, postoperative shoulder balance, pedicle screw

## Abstract

**Background:**

The objective of this study was to evaluate shoulder balance following posterior spinal fusion for thoracic adolescent idiopathic scoliosis (AIS).

**Methods:**

Twenty-four patients (22 females) with thoracic AIS who had undergone posterior fusion with segmental pedicle screws were retrospectively reviewed. The mean follow-up duration was 29 (range, 24–55) months. Fifteen patients had type 1 curves, seven had type 2 curves, and two had type 3 curves according to the Lenke classification. The proximal thoracic (PT) and main thoracic (MT) Cobb angles, percent correction of PT (PTC) and MT (MTC) curves, T1 tilt, and shoulder asymmetry according to radiographic shoulder height (RSH) were measured on preoperative, immediately postoperative, and final follow-up radiographs. The preoperative PT and MT curve side-bending percent correction (PTBC and MTBC) were also measured. The PTC:MTC ratio was employed as an index of PTC and MTC matching. Patients were divided into two groups according to radiographic findings immediately postoperatively: the balanced group (|RSH| <20 mm) and imbalanced group (|RSH| ≥20 mm). The preoperative indices (RSH, PTBC, MTBC, PTC, and MTC), preoperative and postoperative T1 tilt, and PTC:MTC ratio were compared between the two groups.

**Results:**

The mean PT and MT were 33.0° and 64.2° preoperatively, 16.1° (50.5%) and 16.8° (74.0%) immediately postoperatively, and 16.9° (49.0%) and 19.2° (70.3%) at final follow-up, respectively. The mean preoperative RSH of −12.3 mm changed to +11.1 mm immediately postoperatively and improved to +5.7 mm at final follow-up. Seventeen patients were “balanced” and seven were “imbalanced” immediately postoperatively. There were significant differences in the PTC (p=0.04), postoperative T1 tilt (p=0.04), and PTC:MTC ratio (p=0.02) between the two groups (Wilcoxon rank-sum test). Only one patient had an imbalanced shoulder at the final follow-up. She had marked shoulder imbalance immediately postoperatively (RSH: +40 mm).

**Conclusions:**

Sufficient correction of PT curves that is matched with correction of MT curves is necessary to prevent postoperative shoulder imbalance. Almost all patients in our series had satisfactory results in terms of shoulder balance at final follow-up, but one patient with marked shoulder imbalance immediately postoperatively may have residual long-term shoulder imbalance.

## Background

Well-balanced shoulders are important for patient satisfaction after surgery for adolescent idiopathic scoliosis (AIS). In recent years, segmental pedicle screws (SPS) have been used more frequently in posterior spinal fusion for AIS. These constructs ensure better correction of coronal deformities. However, vigorous correction of the main thoracic curve can induce postoperative shoulder decompensation, which can result in shoulder imbalance. The objective of the present study was to evaluate shoulder balance after posterior spinal fusion with SPS constructs for thoracic AIS.

## Methods

Twenty-four patients (22 females) with thoracic AIS who had undergone posterior fusion at a single institution (Osaka City General Hospital) from 2008 through 2011 were retrospectively reviewed. The mean patient age at surgery was 15.8 (range, 12.1–20.5) years. A minimum 2-year follow-up period was required for inclusion in the study. The mean follow-up period was 29 (range, 24–55) months. Based on the Lenke classification [1], fifteen patients had type 1 curves, seven had type 2 curves, and two had type 3 curves.

All patients had undergone surgery using SPS constructs. Pedicle screws were placed in both the convex and concave side of every pedicle in the fusion area with the exception of thin pedicles, screw placement in which is associated with risks of neurologic and vascular complications. When pedicle screw placement in the upper instrumented vertebra (UIV) was difficult, a transverse hook was used instead of a screw. The level of the UIV was determined based on the preoperative flexibility of the proximal thoracic (PT) curve, preoperative shoulder balance, and surgeon’s preference. Consequently, T2, T3, T4, and T5 was selected as the UIV in 1, 12, 8, and 3 patients, respectively. Among seven patients with a double thoracic curve (Lenke type 2), T2, T3, and T4 was selected as the UIV in one, five, and one patient, respectively.

Preoperative evaluation involved examination of whole-spine erect posteroanterior (PA) and supine side-bending radiographs. Cobb angles of the PT curves, main thoracic (MT) curves, T1 tilt, and shoulder asymmetry according to the radiographic shoulder height (RSH) were measured from erect PA radiographs as coronal parameters. RSH was defined as the difference in the soft-tissue shadow directly superior to the acromioclavicular joint [2]. A positive RSH and T1 tilt was defined as left-side up/right-side down. Curve flexibility was evaluated on supine side-bending radiographs. Postoperative evaluation included examination of erect PA radiographs. PT curves, MT curves, T1 tilt, and RSH were measured immediately postoperatively and on radiographs at the final follow-up.

As a measure of preoperative flexibility, preoperative PT and MT curve side-bending correction (PTBC and MTBC, respectively) were calculated using the following formula:

PTBC or MTBC = (preoperative erect Cobb angle − supine side-bending Cobb angle) / preoperative erect Cobb angle × 100%

Postoperative PT correction (PTC) and MT correction (MTC) were calculated using the following formula:

PTC or MTC = (preoperative erect Cobb angle − postoperative erect Cobb angle) / preoperative erect Cobb angle × 100%

The PTC:MTC ratio was defined as an index of the matching of PTC and MTC.

Patients were divided into two groups according to radiographs taken immediately postoperatively: the balanced group (|RSH| <20 mm) and imbalanced group (|RSH| ≥20 mm). Preoperative indices (RSH, T1 tilt, PTBC, and MTBC) were compared with the PTC, MTC, T1 tilt, and PTC:MTC ratio immediately postoperatively between the two groups. The Wilcoxon rank-sum test was used to assess differences between the two groups. A p value of <0.05 was considered statistically significant.

Written informed consent was obtained from the patients for publication of this report and any accompanying images. Ethical approval was not required because this was a retrospective observational study.

## Results

**Figure 1:**
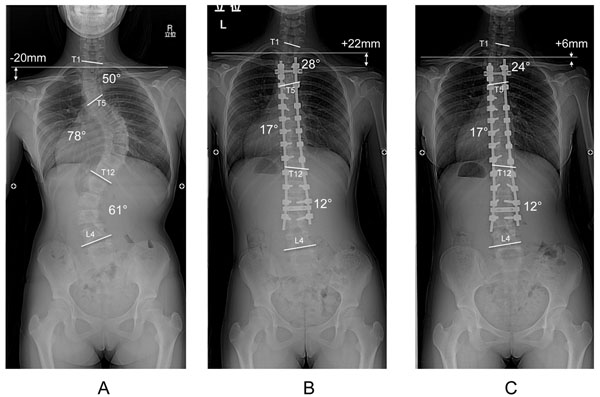
**Fourteen-year-old girl with Lenke type 2C adolescent idiopathic scoliosis.A,** The preoperative Cobb angle of the PT and MT curves were 50° and 78°, respectively. **B,** Immediately after surgery, the PT and MT curves were corrected to 28° (44.0%) and 17° (78.2%), respectively. The PTC:MTC ratio was calculated as 0.56. This patient had postoperative shoulder imbalance (RSH: +22mm). **C,** Five years postoperatively, the RSH had decreased to +6 mm and the postoperative shoulder imbalance had improved. Consent for publication of this figure was obtained from the patient's parents.

The mean preoperative Cobb angle of the PT curve was 33.0° (range, 11°–50°). The PT curve was corrected to 16.1° (range, 3°–28°), and the PTC immediately postoperatively was 50.5% (21.1%–72.7%). At the final follow-up, the mean Cobb angle was 16.9° (range, 2°–27°) and the final PTC was 49.0% (15.8%–81.8%). The mean preoperative Cobb angle of the MT curve was 64.2° (range, 48°–89°). The MT curve was corrected to 16.8° (range, 6°–26°), and the PTC immediately postoperatively was 74.0% (64.9%–90.3%). At the final follow-up, the mean Cobb angle was 19.2° (range, 11°–33°) and the final MTC was 70.3% (56.2%–82.1%). The mean preoperative RSH of −12.3 mm (range, −35 to +20 mm) changed to +11.1 mm (−4 to +40 mm) immediately postoperatively. At the final follow-up, the RSH had improved to +5.7 mm (range, −9 to +36 mm) (Figure 1). Changes in the other preoperative and postoperative radiological parameters, including comparison of single thoracic and double thoracic patterns, are shown in Table 1.

**Table 1:** Clinical results

	All cases	Single thoracic	Double thoracic	P-value*
PT Preop (°)	33.0	28.5	43.9	0.0003

Postop (°)	16.1	14.8	19.3	0.09

Postop PTC (%)	50.5	48.1	56.2	0.14

Final follow-up (°)	16.8	15.1	21.4	0.02

Final follow-up PTC (%)	49.0	48.0	51.2	0.50

MT Preop (°)	49.5	61.1	71.7	0.04

Postop (°)	16.8	16.3	17.9	0.36

Postop MTC (%)	74.0	73.3	75.5	0.25

Final follow-up (°)	19.2	18.9	19.9	0.90

Final follow-up PTC (%)	70.3	69.4	72.5	0.31

RSH Preop (mm)	−12.7	−14.8	−6.3	0.39

Postop (mm)	+11.1	+12.4	+6.7	0.55

Final follow-up (mm)	+5.7	+5.1	+7.1	0.39

T1 tilt Preop (°)	−1.8	−3.4	+2.1	0.01

Postop (°)	+4.8	+3.8	+7.3	0.06

Final follow-up (°)	+3.3	+2.2	+6.0	0.09

PTC:MTC ratio Postop	0.68	0.65	0.74	0.18

*Statistical analysis was performed to compare the single thoracic pattern (Lenke type 1 or 3) with the double thoracic pattern (Lenke type 2) using the Wilcoxon rank-sum test.

Seventeen patients were placed in the balanced group and seven patients were placed in the imbalanced group according to immediately postoperative radiographs. The preoperative RSH was −13.6 mm (range, −35 to +20 mm) in the balanced group and −9.1 mm (−24 to +9 mm) in the imbalanced group. The preoperative T1 tilt was −1.6° (range, −12° to +7°) in the balanced group and −2.0° (−8° to +3°) in the imbalanced group. The PTBC and MTBC were 40.2% (range, 10.5%–72.7%) and 46.7% (29.6%–74.5%) in the balanced group and 46.3% (31.8%–60.0%) and 58.2% (33.8%–76.7%) in the imbalanced group, respectively. Immediately postoperatively, the PTC and MTC were 53.3% (range, 21.1%–72.7%) and 73.2% (64.9%–88.5%) in the balanced group and 43.7% (38.5%–50.0%) and 75.8% (66.7%–90.3%) in the imbalanced group, respectively. Immediately postoperatively, the T1 tilt was +3.6° (range, −3° to +10°) in the balanced group and +7.7° (0° to +12°) in the imbalanced group. The PTC:MTC ratio was calculated as 0.72 (range, 0.32–1.00) in the balanced group and 0.58 (0.45–0.70) in the imbalanced group. There were significant differences in the PTC (p=0.03), postoperative T1 tilt (p=0.04), and PTC:MTC ratio (p=0.02) between the two groups (Table 2).

**Table 2:** Comparison between the balanced and the imbalanced groups

	Balanced group	Imbalanced group	P-value
Preoperative RSH (mm)	−13.6	−9.1	0.19

Preoperative T1 tilt (°)	−1.6	−2.0	0.90

PTBC (%)	40.2	46.3	0.28

MTBC (%)	46.7	58.2	0.08

PTC (%)	53.3	43.7	0.03

MTC (%)	73.2	75.8	0.23

Postoperative T1 tilt (°)	3.6	7.7	0.04

PTC:MTC ratio	0.72	0.58	0.02

There were significant differences in the PTC, postoperative T1 tilt, and PTC:MTC ratio between the two groups (Wilcoxon rank-sum test).

Of the seven patients in the imbalanced group, the shoulder of one patient remained imbalanced at the final follow-up. She had marked shoulder imbalance immediately postoperatively (RSH: +40 mm).

## Discussion

Since the dawn of the Harrington instrumentation era, the necessity of corrective fusion of PT curves has been emphasized to prevent shoulder imbalance after surgical treatment of scoliosis. King et al. [3] reported that a double thoracic curve (defined as T1 tilted into the convexity of the PT curve) should be used to fuse the PT and MT curves with Harrington instrumentation. Lenke et al. [4] stated that a PT curve of >30° that corrected to ≤20° upon side bending, that had grade ≥1 rotation or ≥1-cm translation at the apex of the curve, that showed elevation or a positive T1 tilt, or that had transitional vertebrae between the two curves at T6 or below should be fused when using Cotrel–Dubousset instrumentation. Suk et al. [5] stated that a PT curve of >25° and a level or elevated left shoulder should be treated with fusion of the PT and MT curves if using SPS constructs. The indications for fusion of PT curves have become stricter since the introduction of more powerful spinal instrumentation, which increases the risk of postoperative shoulder decompensation.

The present study showed significant differences in the PTC, postoperative T1 tilt, and PTC:MTC ratio between the balanced and imbalanced groups. Hence, irrespective of whether PT correction is spontaneous or accomplished by instrumentation, sufficient matching of correction of the PT and MT curves is necessary to prevent postoperative shoulder imbalance.

Postoperative shoulder imbalance was chronologically improved in almost all patients during the minimum 2-year follow-up period. However, one patient continued to have an imbalanced shoulder at the final follow-up. Thus, patients with marked shoulder imbalance immediately postoperatively may have long-term residual shoulder imbalance. Additionally, Cao et al. [6] reported RSH to be significantly correlated with the parameters of distal adding-on. Therefore, avoiding shoulder imbalance immediately postoperatively should be emphasized for favorable long-term patient satisfaction and operative outcomes.

## Conclusions

Sufficient correction of PT curves matched to correction of MT curves is necessary to prevent postoperative shoulder imbalance. Almost all patients in our series had satisfactory outcomes in terms of shoulder balance at the final follow-up. However, avoiding shoulder imbalance immediately postoperatively is important because marked shoulder imbalance immediately postoperatively could induce residual shoulder imbalance in the long term.

This study was presented at the 10^th^ Meeting of the International Research Society of Spinal Deformities (IRSSD 2014 Sapporo) [7].

## Competing interests

The authors declare that they have no competing interests.

## Authors' contributions

TN made substantial contributions to the study design, analysis and interpretation of the data, and drafting of the manuscript. AM and MK performed the surgical procedures, obtained the radiographs, and collected the data. KH collected the data. HN coordinated the project. All authors read and approved the final manuscript.

## Declarations

Publication charges for this collection were funded by IRSSD 2014 Sapporo.

This article has been published as part of *Scoliosis* Volume 10 Supplement 2, 2015: Research into Spinal Deformities 9: Short Papers from the IRSSD 2014 Meeting. The full contents of the supplement are available online at http://www.scoliosisjournal.com/supplements/10/S2.
